# Early onset of graft glomerulopathy in a patient with post-transplant diabetes mellitus after renal transplantation: a case report

**DOI:** 10.1186/s12882-018-1141-9

**Published:** 2018-12-07

**Authors:** Marilena Gregorini, Vincenzo Sepe, Francesca Eleonora Pattonieri, Anna Allesina, Teresa Rampino

**Affiliations:** 1 Nephrology, Dialysis and Renal Transplant Unit, IRCCS Policlinico San Matteo Foundation, P.le Golgi 19, 27100 Pavia, Italy; 2 Department of Internal Medicine and Therapeutics, University of Pavia, Via Aselli 43/45, 27100 Pavia, Italy; 3 PhD in Experimental Medicine, University of Pavia, Via Forlanini 6, 27100 Pavia, Italy

**Keywords:** PTDM, Kidney transplantation; diabetic nephropathy, Mesangiolysis, CNI, Microalbuminuria, HbA1c, mTOR inhibitors

## Abstract

**Background:**

Post-transplant diabetes mellitus (PTDM) is an emerging problem in kidney transplantation, representing an important risk factor for kidney function loss. Diabetic nephropathy (DN) occurrence in transplanted kidneys is poorly investigated. Current knowledge describes DN recurrence in graft 5.9 years from kidney transplantation however there is little data about PTDM and DN.

Here, we report a clinical case peculiar for an early appearance of advanced glomerular diabetic lesions, after kidney transplantation.

**Case presentation:**

A 45-year-old Caucasian male affected by autosomal polycystic kidney disease was transplanted with a cadaveric-kidney-donor from 58-year-old male. Induction immunosuppressive therapy included basiliximab and steroids while the maintenance treatment included, tacrolimus, mofetil micophenolate and methylprednisolone.

One month after transplantation the patient developed diabetes requiring treatment with repaglinide quickly replaced with insulin to obtain an acceptable glycemic control (HbA1c 52 mmol/mol). Glycosuria was detected persistently during the first six months after transplantation. To achieve further improvement in glycemic control, a shift from tacrolimus to cyclosporine (CyA) was made and steroids were rapidly tapered and stopped. To minimize calcineurin inhibitors toxicity, which was revealed in the 1-year-protocol-biopsy, everolimus was introduced thereby lowering CyA through levels. Moderate hypertension was well controlled with doxazosin. Thirty months after transplantation a second graft biopsy was performed owing to renal function decline and microalbuminuria appearance. Histological analysis surprisingly showed mesangiolysis and microaneurysms; glomerular sclero-hyalinosis and basal membrane thickness and typical nodular glomerulosclerosis. C4d staining was negative and no evidence of immune deposits were detected. Donor Specific Antibodies, serum C3 and C4 levels and autoimmunity tests were negative. Retrospective analysis on donor history didn’t show diabetes or insulin resistance and no diabetic lesions were found in kidney pre-implant biopsy.

**Conclusions:**

In our knowledge, this is the first report describing a very early onset of advanced diabetic glomerular lesions in a graft biopsy after PTDM. We hypothesize that additional factors such as everolimus and hypertension, may have contribute to kidney damage.

**Electronic supplementary material:**

The online version of this article (10.1186/s12882-018-1141-9) contains supplementary material, which is available to authorized users.

## Background

Post-transplant diabetes mellitus (PTDM) is a metabolic complication following renal transplant whose incidence ranges between 4 and 25%. Often the rapid onset and the accelerated course of diabetic nephropathy (DN) in post transplantation, if not recognized promptly, can have serious consequences. So a multidisciplinary approach in post transplantation and the endocrinologist’s role in these patients are crucial. PTDM exhibits similar complications to those seen in patients with type II diabetes, but with an accelerated rate, which can worsen the outcomes of transplant including graft failure and death [1].

Areas of mesangiolysis with glomerular capillary microaneurysm are seen in native kidneys after several years of diabetes and these are the hallmark of advanced diabetic DN.

We report an interesting case of advanced diabetic lesions such as mesangiolysis, microaneurysm and nodular glomerulosclerosis observed in a kidney biopsied after only 30 months from transplantation.

To the best of our knowledge, this is the first case reporting an early occurrence of advanced diabetic lesions in PTDM after kidney transplant.

## Case presentation

One month following cadaveric kidney transplantation a 45-year-old Caucasian man, under tacrolimus, micophenolate mofetil (MMF) and steroids immunosuppression developed PTDM. Thirty months after transplantation histological graft changes characterized by mesangial sclerosis, mesangiolysis with glomerular capillary ectasia and microaneurysms appeared (Additional file 1). Diagnostic criteria for PDTM were consistent with current American Diabetes Association (ADA) clinical practice recommendation to diagnose diabetes in the general population [2].

**Fig. 1:**
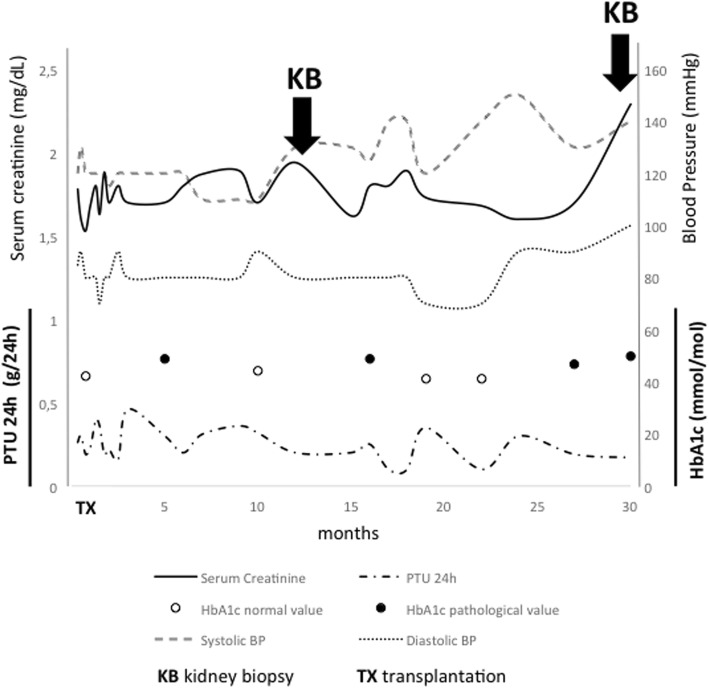
Clinical and biochemical timeline of case report

**Fig. 2:**
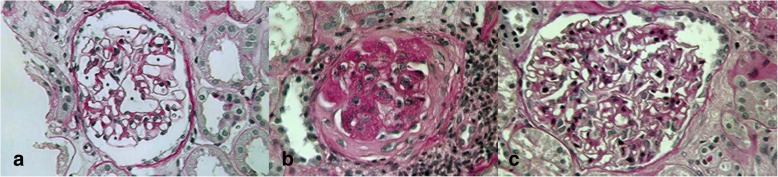
Kidney graft biopsy. Panels a and b: biopsy performed 30 months after renal transplantation and (periodic acid-Schiff staining, magnification × 400). Panel **a**: focal mesangiolysis and dilatated capillary aneurysms (*). Panel **b**: nodular glomerulosclerosis. Panel **c**: donor pre-implant kidney biopsy showing little mesangial cell hyperplasia (periodic acid-Schiff staining, magnification × 400)

The deceased donor was a 58-year-old man whose cause of death was cerebral hemorrhage. The cold storage time was 12 h. The recipient’s cause of end stage renal disease (ESRD) was autosomal polycystic kidney disease. Non-modifiable recipient risk factors for diabetes were, excluding polycystic kidney disease, male gender and family history for diabetes, while donor non-modifiable risks included male gender, deceased donor. The donor history was negative for diabetes or insulin resistance. A week after transplantation, the recipient developed a moderate hypertension (Fig. 1) requiring doxazosin treatment resulting in good blood pressure control. The patient‘s BMI was always within normal range values. Basiliximab was used as induction therapy. The maintenance immunosuppressive regimen included tacrolimus, MMF, and methylprednisolone. To treat the PTDM, repaglinide was introduced with a poor glycemic control; consequently it was substituted with insulin followed by acceptable glycemic levels: HbA1c 6.7% (52 mmol/mol). At time of PTDM diagnosis creatinine serum level (SCr) was 1.5 mg/dL and 24-h proteinuria was 300 mg (Fig. 1). Glycosuria was detected persistently in the first six months after transplantation and the basal glucose monitoring showed glycemia levels that didn’t exceed 180 mg/dL. To achieve further improvement in diabetes management, tacrolimus was replaced with cyclosporine (CyA). Steroids were rapidly tapered and stopped. To minimize calcineurin inhibitors (CNI) toxicity, which was revealed in a 1-year-protocol biopsy, everolimus was introduced thereby lowering CyA through levels. A mild mixed dyslipidemia was controlled with atorvastatin and omega-3 fatty acid. A second graft biopsy was performed 30 months after transplantation due to renal function decline, SCr was 2.2 mg/dL, 24-h 200 mg microalbuminuria appeared, and BMI was 21. Although treatment with doxazosin and bisoprolol were titrated at each follow-up control, blood pressure always ranged between 140 and 160/90–100 mmHg (Fig. 1). The patient did not accept angiotensin-converting enzyme inhibitors (ACE-I) and/or angiotensin II receptor blockers (ARBs) because of associated side effects, cough and erectile dysfunction respectively. The main clinical and laboratory data are shown in Fig. 1. At this point Donor Specific Antibodies, serum C3 and C4 levels and autoimmunity tests were negative, microalbuminuria/creatininuria ratio was 56.82 mg/g. Histological analysis surprisingly showed mesangiolysis, glomerular capillary ectasia and microaneurysms (Fig. 2 panel a). It was observed in about 30% of glomeruli; moreover sclero-hyalinosis, basal membranes thickness in 20% of glomeruli and typical nodular glomerulosclerosis as showed in Fig. 2 panel b, in few glomeruli. C4d staining was negative and no evidence of immune deposits was detected. According to our post-transplant biopsy protocol, sample collection for electronic microscopy was not performed. After transplantation glycosylated hemoglobin never exceeded 6.9% (52 mmol/mol). A serial Ultrasound Doppler performed on kidney transplant every six months showed Renal Resistive Index (RRI) that didn’t exceed 0.65. A retrospective re-analysis of pre-implant donor biopsy confirmed no diabetic injury (Fig. 2 panel c).

## Discussion and conclusions

Post transplant diabetes is an emerging problem in solid organ transplantation. Most of the anti-rejection drugs used such as glucocorticoids, tacrolimus and sirolimus are burdened by a diabetogenic effect. The prevailing factors causing diabetes by steroids seem to be the aggravation of insulin resistance, defects of insulin secretion and beta-cell apoptosis, while a decline in beta-cell survival and an impairment of insulin secretion are described as mechanism operated by CNI and sirolimus [1].

Nevertheless, the wide use of these drugs, especially tacrolimus, is justified by their powerful immunosuppressive effect.

While a great prevalence of PTDM is observed, not all the diabetic patients experience DN. Additionally, DN is not fully explained by poor glycemic control. Albeit the pathways that lead to DN in the native kidney have been extensively explored, there is still a paucity of data on DN after kidney transplantation [1].

Intrinsic susceptibility to diabetes-related damage in allograft kidneys could be the answer to several factors. Genetic predisposition is an important one; certain human leukocyte antigen (HLA) phenotypes, notably A30, B8, B15, B27, B42, and DR4, are associated with DN, but in our case neither donor nor recipient had HLA-predisposing phenotypes. The only familial factor risk was that the recipient’s mother became diabetic in old age.

Obesity is a known relevant contributor of glomerulosclerosis, but our patient always showed normal BMI values.

The reduced number of glomeruli per kidney has been suggested as another possible individual risk factor for the rapid progression of DN. Even though the donor and recipient were similar in size, the donor was 13 years older than the recipient, this fact could explain a potential reduced number of undamaged glomeruli. Broad evidence supports the association between low birth weight, premature childbirth and lower nephron number; unfortunately there is no such data available for our donor, consequently we cannot with confidence evaluate the equity of glomeruli [1].

In addition diabetes leads to severe impairment of renal vasomotor capacity, leaving the glomeruli unprotected by systemic high-pressure levels [3]. The resulting glomerular over-perfusion may justify the mesangiolysis and glomerular microaneurysm formation, observed in the patient. Experimental models confirm our evidence, showing a link between hypertension, mesangiolysis, endothelial cell loss and glomerular capillary dilatation [4].

Although published clinical evidences show the benefits of ACE-I and/or ARBs on DN progression in native kidneys, limited information exists regarding the benefit of therapeutic interventions, e.g. ACE-I or ARBs, on reducing proteinuria or microalbuminuria after transplantation or PTDM nephropathy and kidney graft survival [5, 6].

In the transplanted patients we need to consider other probable mesangiolysis triggers like acute vascular CNI toxicity, immune-mediated glomerulonephritis and antibody-mediated rejection. However, serological and histological tests performed in the patient, ruled out those causes. The advanced stage of glomerulosclerosis can be justified even in the absence of immune deposits as demonstrated in previous published cases [7].

The anti-proliferative effect of mTOR inhibitors may negatively impact the glomerular repair, lead by growth factors, after mesangiolysis as was observed in the animal models [8, 9]; however in humans it is still an open issue. For the lack of evidence against everolimus, we didn’t plan the drug withdrawal in our patient.

The rapid recurrences of DN after kidney transplantation has been published, but in all cases the patients were already diabetic [7]. The detection of such serious and advanced lesions after only 30 months from PDTM, as described in this case report, is a novelty.

Interestingly, mesangiolysis occurred very early after PTDM even in the presence of controlled glycemia. An HbA1c level < 7% (54 mmol/mol) is a reasonable goal for many patients but it’s not enough in those where hypertension is associated. Patients with PTDM should be strictly followed for microalbuminuria, glycemic and pressure control to minimize the risk of DN onset. Renal biopsy should drive the therapeutic changes and provide new insights into disease knowledge. In the presence of DN it would be recommended to correct all diabetes modifiable risk factors including a controlled lipid profile, no smoking and a healthy life-style. A careful evaluation of immunosuppressive therapy using non-diabetogenic drugs is important.

In conclusion, reducing the dose or discontinuing CNI and steroids could potentially limit the damage to beta cell, although the clinical evidence is poor. However it is difficult to weigh the risks related to rejection and/or PTDM in individual patients to choose the best immunosuppressive medication regimen**.**

Future perspective will evaluate whether belatacept, a new immunosuppressive drug with higher affinity to podocyte B7–1 antigen, expressed on hyperglycemia damaged podocytes, will be effective in preventing the development of DN after kidney transplantation.

## Additional file

Additional file 1: Clinical events timetable from pre-implant kidney biopsy to the post-transplant diabetes mellitus renal histological changes observed. (PPTX 68 kb)

## Abbreviations

ACE-I: Angiotensin-Converting Enzyme Inhibitors
ADA: American Diabetes Association
ARBs: Angiotensin II Receptor Blockers
BMI: Body Mass Index
CNI: Calcineurin Inhibitors
CyA: Cyclosporine
DN: Diabetic Nephropathy
ESRD: End Stage Renal Disease
HbA1c: Glycated Haemoglobin
HLA: Human Leukocyte Antigen
MMF: Micophenolate Mofetil
mTOR: mammalian Target Of Rapamycin
PTDM: Post-Transplant Diabetes Mellitus
RRI: Renal Resistive Index
SCr: Serum Creatinine
