# Supplementary material for: Early onset of graft glomerulopathy in a patient with post-transplant diabetes mellitus after renal transplantation: a case report
Source: BMC Nephrol. 2018 Dec 7;19:348. doi: 10.1186/s12882-018-1141-9 (PMC6286527; doi:10.1186/s12882-018-1141-9)
Supplement: Supplementary file 1 — Clinical events timetable from pre-implant kidney biopsy to the post-transplant diabetes mellitus renal histological changes observed. (PPTX 68 kb) [file 12882_2018_1141_MOESM1_ESM.pptx]

## Slide 1
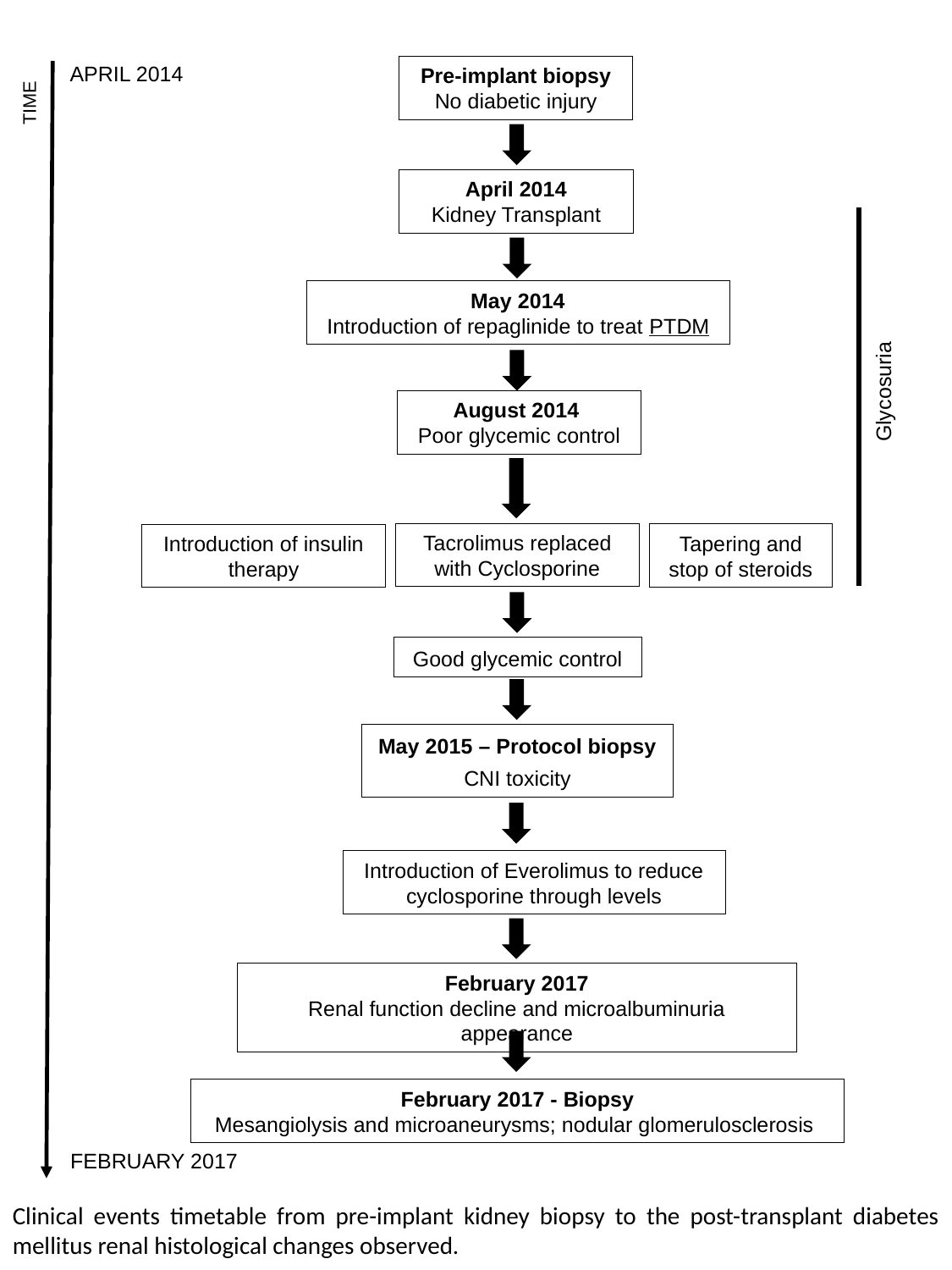

APRIL 2014
Pre-implant biopsy
No diabetic injury
TIME
April 2014
Kidney Transplant
May 2014
Introduction of repaglinide to treat PTDM
Glycosuria
August 2014
Poor glycemic control
Tacrolimus replaced with Cyclosporine
Tapering and stop of steroids
Introduction of insulin therapy
Good glycemic control
May 2015 – Protocol biopsy
CNI toxicity
Introduction of Everolimus to reduce cyclosporine through levels
February 2017
Renal function decline and microalbuminuria appearance
February 2017 - Biopsy
Mesangiolysis and microaneurysms; nodular glomerulosclerosis
FEBRUARY 2017
Clinical events timetable from pre-implant kidney biopsy to the post-transplant diabetes mellitus renal histological changes observed.
